# Mannose-Binding Lectin Codon 54 Gene Polymorphism and Vulvovaginal Candidiasis: A Systematic Review and Meta-Analysis

**DOI:** 10.1155/2014/738298

**Published:** 2014-01-06

**Authors:** Bojan Nedovic, Brunella Posteraro, Emanuele Leoncini, Alberto Ruggeri, Rosarita Amore, Maurizio Sanguinetti, Walter Ricciardi, Stefania Boccia

**Affiliations:** ^1^Institute of Public Health, Section of Hygiene, Università Cattolica del Sacro Cuore, 00168 Rome, Italy; ^2^Institute of Microbiology, Università Cattolica del Sacro Cuore, Largo F. Vito 1, 00168 Rome, Italy; ^3^Unit of Clinical and Molecular Epidemiology, IRCCS San Raffaele Pisana, 00167 Rome, Italy

## Abstract

Mannose-binding lectin (*MBL*) plays a key role in the human innate immune response. It has been shown that polymorphisms in the *MBL2* gene, particularly at codon 54 (variant allele *B*; wild-type allele designated as *A*), impact upon host susceptibility to *Candida* infection. This systematic review and meta-analysis were performed to assess the association between *MBL2* codon 54 genotype and vulvovaginal candidiasis (VVC) or recurrent VVC (RVVC). Studies were searched in MEDLINE, SCOPUS, and ISI Web of Science until April 2013. Five studies including 704 women (386 cases and 318 controls) were part of the meta-analysis, and pooled ORs were calculated using the random effects model. For subjects with RVVC, ORs of *AB* versus *AA* and of *BB* versus *AA* were 4.84 (95% CI 2.10–11.15; *P* for heterogeneity = 0.013; *I* ^2^ = 68.6%) and 12.68 (95% CI 3.74–42.92; *P* for heterogeneity = 0.932, *I* ^2^ = 0.0%), respectively. For subjects with VVC, OR of *AB* versus *AA* was 2.57 (95% CI 1.29–5.12; *P* for heterogeneity = 0.897; *I* ^2^ = 0.0%). This analysis indicates that heterozygosity for the *MBL2* allele *B* increases significantly the risk for both diseases, suggesting that MBL may influence the women's innate immunity in response to *Candida*.

## 1. Introduction

Vulvovaginal candidiasis (VVC) and recurrent vulvovaginal candidiasis (RVVC), both caused by overgrowth of *Candida* species, especially *Candida albicans*, remain a significant problem worldwide [1]. It is estimated that 75% of all women experiences VVC at least once during their lives, and about half of them have at least one recurrence [2]. Moreover, up to 8% of these women will suffer from RVVC [3], which consists in frequent individual attacks of acute, symptomatic VVC [4].

Different components of the innate immune system, such as the Toll-like receptors (TLRs) and C-type lectins (CLRs), play a major role in the recognition of molecular patterns on the *C. albicans* cell wall, leading to phagocytosis and killing of the invading fungus [5]. This also interacts with the vaginal epithelium that represents *per se* an important innate anti-*Candida* defense through the expression of the CLR dectin-1 and several TLRs [5]. Nonetheless, various genetic changes have been identified that affect these immune mediators and, consequently, influence the innate immunity in response to *Candida*, thus increasing the host susceptibility to (R)VVC [6].

Among CLRs, the soluble, opsonic, multimeric mannose-binding lectin (*MBL*) is able to trigger the complement cascade by recognizing and binding to carbohydrate moieties on the surface of microorganisms in general [7, 8] and *C. albicans *in particular [9, 10]. Single nucleotide polymorphisms (SNPs), Gly54Asp (rs1800450), Gly57Glu (rs1800451), and Arg52Cys (rs5030737) in exon 1 of the *MBL2* gene result in three different allelic variants (*B*, *C*, and *D*, resp.; wild-type denominated as *A*), which lead to nonfunctional monomers in homozygotes or reduce the amount of functional monomers in heterozygotes [11]. As the effect on serum *MBL* is rather similar, the structural *MBL2* gene variants *B*, *C*, and *D *are often pooled and referred to collectively as *O* [11]. In addition, three *MBL2* gene promoter polymorphisms, G-550C (*H*/*L*), G-221C (*Y*/*X*), and C+4T (*P*/*Q*), have been described, giving rise to combinations that are associated with different levels of *MBL* protein, such as high (*HY*), intermediate (*LY*), and low (*LX*) levels [12]. As linked to the independent *B*, *C*, and *D* SNPs, these combinations form seven common “secretor haplotypes” that ultimately define the serum *MBL* concentrations, that is, high with the *HYPA*, *LYQA*, and *LYPA* haplotypes and low with the *LXPA*, *HYPD*, *LYPB*, and *LYQC* haplotypes [13].

As most frequent in healthy Caucasians [11], the *MBL2* codon 54 polymorphism has been associated with reduced vaginal concentrations of *MBL* [14, 15] and with an increased rate of RVVC in Latvian, Brazilian, Chinese, and Belgium [14, 16–18] but not Italian [19] patients. Moreover, allele *B* was also more frequent in women with recurrent bacterial vaginosis [16] as well as women with a single episode of symptomatic VVC [17], suggesting that this polymorphism may not be the sole determinant of susceptibility to RVVC. Nevertheless, higher *MBL* serum levels were found in women suffering from RVVC than in healthy women [20], indicating that *MBL* may be effective in the acute-phase defense against this condition [21].

To attempt to clarify this matter, therefore, we performed a systematic review and meta-analysis to determine whether the presence of *MBL* codon 54 gene polymorphism enhances the risk of (R)VVC in adult women. We listed all the case-control studies to show the evidence in the literature on the association of the variant allele *B* carriage with the occurrence of vulvovaginal *Candida* infection.

## 2. Methods

This paper was undertaken according to the Preferred Reporting Items for Systematic Reviews and Meta-Analyses (PRISMA) guidelines [22].

### 2.1. Search Strategy and Inclusion Criteria

The electronic databases MEDLINE, SCOPUS, and ISI Web of Science (since initiations to 30 April 2013) were searched for relevant studies using the following terms: mycosis AND (“*MBL* gene polymorphism” OR “*MBL2* gene polymorphism” OR “protein C” or “mannose-binding lectin”). All searches were limited to human subjects without language, study design, or publication status restrictions. The reference lists of retrieved articles were also screened to find relevant articles that were not identified by the initial search strategy. Moreover, the MEDLINE was searched monthly after April 2013 to identify any newly published studies. Searching was performed by two independent reviewers (BN and AR).

Any study was considered to be eligible for inclusion in our systematic review and meta-analysis if it met the following criteria: (i) the *MBL* gene polymorphism at codon 54 was determined; (ii) the outcome was VVC or RVVC, and there were at least two comparison groups, for example, (R)VVC versus control groups (subjects with no history of vaginal *Candida* infection and/or clinical symptoms of VVC); (iii) frequencies of alleles *B* (variant) and *A* (wild-type) were assessed in both cases and controls; (iv) the allele *B* frequency in the control group did not deviate from the Hardy-Weinberg equilibrium (HWE) at *P* ≤ 0.01; and (v) participants were nonpregnant women.

### 2.2. Data Extraction

Data were extracted independently by two reviewers (BN and AR) who developed a customized database for data extraction. For each study, the following information was collected: first author, year and location of the study, average age, ethnicity, number of participants, number of cases and controls, and frequency of the *MBL2* genotypes in cases and controls. To allow appropriate comparison of all studies, cases and controls were classified as *AA* (*MBL2* codon 54 wild-type genotype), *AB* (*MBL2* codon 54 heterozygous genotype), or *BB* (*MBL2* codon 54 homozygous genotype). Any disagreement was settled by consensus between the two reviewers. If no agreement could be reached, it was solved through discussion with a third reviewer (SB).

### 2.3. Quality Assessment

Two reviewers (BN and BP) independently examined the quality of each included study through a checklist modified from a previously published meta-analysis of molecular association studies, using a risk of bias score based on both epidemiologic and genetic issues [23]. Six domains were assessed, that were representativeness of cases, representativeness of controls, ascertainment of (R)VVC, ascertainment of controls, genotypic examination, and association assessment. Disagreements between the two reviewers were solved by the third reviewer.

### 2.4. Outcomes of Interest

The outcomes of interest were VVC and RVVC, which were defined according to original studies. Briefly, RVVC was diagnosed if women had four or more culture-documented episodes of VVC during a 12-month period before enrolment. VVC was diagnosed if women had symptoms consistent with a vaginal *Candida* infection together with a mycological culture positive for *Candida* species.

### 2.5. Statistical Analysis

The odds ratios (ORs) with 95% confidence intervals (CIs) were calculated for the meta-analysis. A random effects model, using the reciprocal of the variance as the weighting factor, was applied throughout to provide a more conservative estimate of the effect size and to take into account between-study variation [24]. Statistical heterogeneity across studies was quantified with the *I* ^2^ lying between 0% and 100%, where values less than 40% suggest that homogeneity is good for the reliability of meta-analysis [25]. To evaluate the weight each study had on the overall estimate, Galbraith's test was performed as previously described [24]. To further explore the reasons of heterogeneity, we conducted sensitivity analyses by omitting each study one at a time. Deviation from HWE in each study was determined according to previous studies [24]. All analyses were performed using STATA version 12.0 software and a two-sided *P* value less than 0.05 was considered statistically significant.

## 3. Results

**Figure 1:**
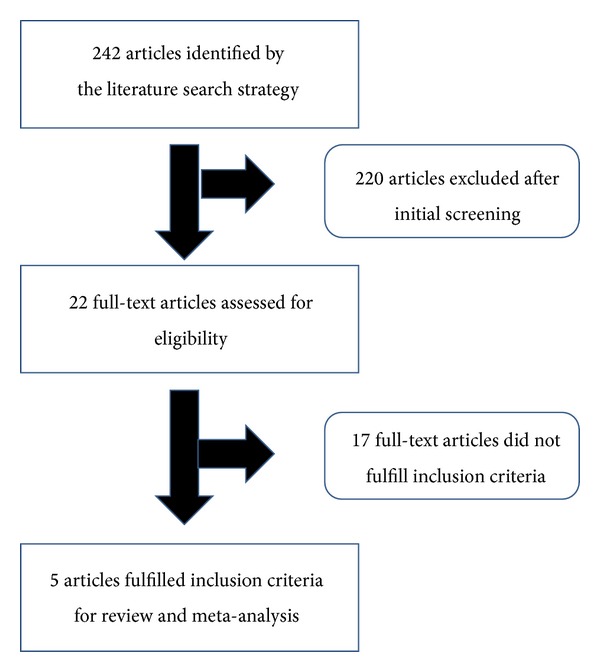
Flow chart of the study selection.

Our initial literature search yielded a total of 242 references, of which 70 from MEDLINE, 151 from SCOPUS, and 21 from ISI Web Science. After screening the titles and abstracts, 220 studies were excluded, because they were not considered relevant to the study topic or because of duplication in the searched databases, leaving 22 potentially eligible studies. After reading the full texts, 17 studies were discarded because they did not meet the above-specified inclusion criteria, and only 5 studies [14–18] were used for data extraction. Our subsequent search did not yield any additional study, and no relevant studies were published after April 2013. A flow chart showing the study selection is given in Figure 1.

Characteristics of each case-control study included in our meta-analysis are summarized in Table 1. Two studies were conducted in South America and the rest (1 study each) in Europe, Latvia, and China, with a total of 704 women involved. The average age of participants across the cases (*n* = 386) ranged from 27 to 36 years and across the controls (*n* = 318) from 25 to 33 years. The nonpregnancy was one of the inclusion criteria for all the studies, followed by the absence of diabetes mellitus, immunodeficiency, or use of immunosuppressive medications for 2 of 5 studies [14, 15]. All of the cases were diagnosed as RVVC (*n* = 307) or VVC (*n* = 79) according to standard diagnostic guidelines, whereas all of controls were matched to the cases at least for age. Genotype frequencies in all of the control groups did not deviate from values predicted by HWE (data not shown).

The study quality was satisfactory overall, and the selected studies had low risk of bias from ascertainment of (R)VVC and the *MBL2* allele genotypes, while the description of controls was clearly accessible in 2 of 5 (40%) studies [14, 18], so bias from control selection might be present in the remaining 3 studies [15–17].

**Figure 2:**
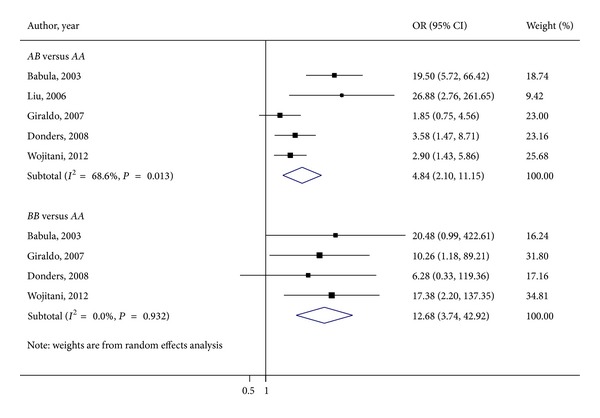
Meta-analysis of the influence of *MBL2* genotype on RVVC susceptibility: *AB* versus *AA* and *BB* versus *AA* forest plots.

All of the 5 studies included in our meta-analysis have reported association between *MBL2* codon 54 polymorphism and RVVC.  Figure 2 shows the ORs for vaginal *Candida* infection comparing subjects with *AA* genotypes and those with *AB* or *BB* genotypes. Applying the random effects model yielded the pooled ORs for *AB* versus *AA* or* BB *versus *AA* of 4.84 (95% CI 2.10–11.15; *P* for heterogeneity = 0.013; *I* ^2^ = 68.6%) and 12.68 (95% CI 3.74–42.92, *P* for heterogeneity = 0.932, *I* ^2^ = 0.0%), respectively. As the OR for *AB* versus *AA* was shown to be highly heterogeneous, this finding was explored by means of Galbraith's test and, then, we singled out the study of Babula et al. [14] as a main contributor to heterogeneity (data not shown). Furthermore, when the same study [14] was omitted based on the sensitivity analysis, we found that the OR for *AB* versus *AA* was 3.16 (95% CI 1.69–5.9; *P* for heterogeneity = 0.849; *I* ^2^ = 38.0%). Taken together, our results suggested that women carrying *AB* or *BB *genotypes had 5 times and 13 times higher odds of RVVC than those carrying the *AA* genotype.

**Figure 3:**
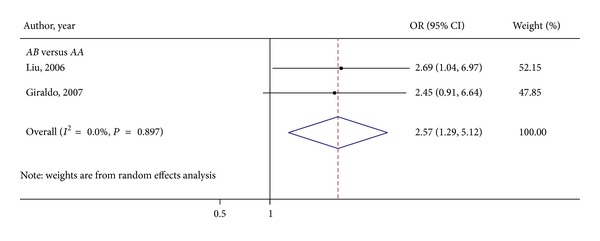
Meta-analysis of the influence of *MBL2* genotype on VVC susceptibility: *AB* versus *AA *forest plot.

Two of the 5 studies included in our meta-analysis reported association between *MBL2* codon 54 polymorphism and VVC [16, 17].  Figure 3 shows the ORs for vaginal *Candida* infection comparing subjects with *AA* genotypes and those with *AB* genotypes, because of the lack of homozygous *BB* genotype subjects across the selected studies. Applying the random effects model yielded the pooled OR for *AB* versus *AA* of 2.57 (95% CI 1.29–5.12; *P* for heterogeneity = 0.897; *I* ^2^ = 0.0%), suggesting that women carrying the *AB* genotype had 2-time higher odds of VVC compared with those carrying the *AA* genotype.

## 4. Discussion

This meta-analysis of accessible, published data derived from 5 studies considering cases of RVVC and/or VVC showed that *MBL* codon 54 gene polymorphism is significantly associated with both the fungal diseases. In particular, possessing the *MBL* variant allele *B* heterozygous genotype increases the susceptibility of women to RVVC or VVC compared to healthy controls, while the risk of RVVC is also increased for women carrying the allele *B* homozygote genotype. These results suggest that heterozygosity for the *MBL2* allele *B*, causing an *MBL* structural defect [11], confers a greater protective effect than homozygosity for the same allele.

Although genotypic analyses can be used as surrogates for *MBL* serum levels [26], it was recently speculated that the genotype and the serum level for *MBL2* may have different clinical implications [27]. As the acute-phase reactant, liver protein *MBL* is thought to transudate from the blood circulation into the female genital tract, this could account for the level variations seen in vulvovaginal fluids according to the individual's *MBL2* genotypes [14]. It is also likely that vaginal epithelial cells may themself contribute to the *MBL* concentrations [14].

In one study [14], almost 62% of patients with RVVC was allele *B* heterozygotes, and only 3% of patients was allele *B* homozygotes, but not all the RVVC women who had a reduced level of *MBL* in their vaginas did carry the codon 54 variant allele. Thus, if deficient vaginal levels of *MBL* are argued to contribute to vaginal *Candida* proliferation in women with RVVC, unfortunately in that study, no women with a first episode of acute VVC were included to rule out that *MBL* deficiency is a general feature of vulvovaginal *Candida* infection [14]. In another study [17], vaginal wild-type or heterozygous *MBL* concentrations were higher in women with VVC (47 of 51) than in controls, but not in women with RVVC (6 of 6) suggesting that *MBL* may increase during a first attack of *Candida *vaginitis but that subsequent attacks decrease the immune system susceptibility to the fungus.

However, if low MBP concentrations would lead to impaired opsonophagocytosis of *Candida* at the vaginal mucosa surface, it is also conceivable that one patient with a wild-type allele for the *MBL2 *54 polymorphism can have a promoter combination (e.g., the *LX* haplotype) that down-regulates MBP, thereby expressing low levels of functional protein. On the other hand, the *MBL2* structural gene variants alone could be responsible for diminished binding of the *MBL* protein to *Candida* cells via its lectin domain, at the early phase of defense against *Candida* during vaginal infection by the fungus [9]. Nevertheless, none of studies in the present review have examined the association of a combined effect of *MBL* structural and promoter gene polymorphisms with the occurrence of VVC or RVVC, as well as the role of multiple genetic factors in determining the increased susceptibility to RVVC [5].

**Figure 4:**
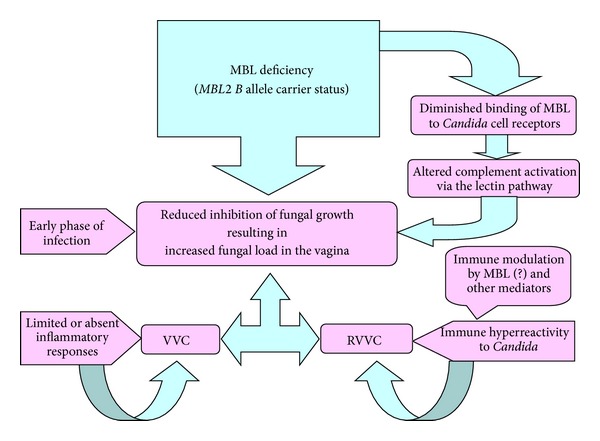
A possible link between the carriage of *MBL* codon 54 gene polymorphism and the host predisposition to either VVC or RVVC.

As resistance and tolerance are complementary host antifungal defense mechanisms that are likely operating in the vaginal mucosa [28], a balanced *MBL* polymorphism would confer some relative benefits to the host, particularly women in the reproductive age when innate immune mechanisms are relatively more important than adaptive immune mechanisms [29]. In this sense, a dual role of *MBL*, both protective and detrimental, in the vaginal environment, could be envisaged to take into account the susceptibility to and the type of outcome (i.e., VVC or RVVC) of *Candida* infection. As opposed to the primary RVVC, an idiopathic form with no known predisposing factors [30], acute VVC or secondary RVVC is associated with several exogenous factors [4]. These factors are supposed to modulate the susceptibility to *Candida* infection, despite the natural protective immune mechanisms which are engaged to limit fungal burden and inflammation at the vaginal mucosa level [4] and, on the other hand, to tolerate a commensal microorganism, like *C. albicans* [29]. Lastly, a deficiency of tolerance, perhaps through the cooperation of immune mediators other than *MBL* [28, 31, 32], could influence the women's likelihood of developing recurrent episodes of VVC. A final hypothesis as to how the *MBL2 B* allele carrier status may predispose to either VVC or RVVC is schematized in Figure 4.

Some limitations of the present meta-analysis should be considered. First, although the review was carried out following rigorous analytical methods and thus biases were due to the selection of studies and less likely due to data extraction, we believe that the quality of the review may have been affected by the fact that only 5 studies were evaluable for the meta-analysis. Second, some of the included studies contained small numbers of cases, including a very low number of RVVC cases, and the backgrounds of patients varied across the included studies, particularly in the two studies involving white and nonwhite Brazilian patients. All of this would result in the lack of power for detection of gene effects, thus requiring further updated meta-analysis provided that more studies are published in the literature. Third, adjusting our estimates for potential confounding factors, such as the well-known risk factors for mucosal *Candida* infection, should lead to more valid pooling results, though there was no sufficient information to this regard. Finally, despite the record of moderate heterogeneity for the pooled estimates, we believe that this meta-analysis could significantly contribute to the understanding of the pathogenesis of (R)VVC.

## 5. Conclusion

In summary, our results showed that women carrying the *B* allele of *MBL* polymorphism might have more risk of developing vulvovaginal candidiasis. As RVVC is likely polygenic and/or multifactorial, further studies with larger sample sizes are required to confirm the role of *MBL2* polymorphisms in association with *Candida* vulvovaginitis. As RVVC may be virtually untreatable, a better deciphering of anti-*Candida* host defense mechanisms in the vagina is crucial to design novel immunotherapeutic strategies in order to optimize and/or replace conventional antifungal treatments.

## Figures and Tables

**Table 1:** Characteristics of case-control studies which are included in meta-analysis.

First author, year of publication	Country	Ethnicity	Participant origin	Average age^a^ of		Sample size (cases/controls)	No. of genotypes (cases/controls)		
				Cases	Controls		*AA *	*AB *	*BB *
Studies describing association between MBL codon 54 gene polymorphism and RVVC									
Babula, 2003 [14]	Latvia	Latvian	Hospital outpatient service	27 (18–35)	25 (18–35)	42/43	13/39	26/4	3/0
Liu, 2006 [17]	China	Chinese	Hospital outpatient service	29 ± 6	31 ± 5	6/54^b^	1/43	5/8	0
Giraldo, 2007 [16]	Brazil	White/nonwhite	Hospital outpatient service	31 (18–68)^c^	33 (16–70)	50/66	31/53	13/12	6/1
Donders, 2008 [18]	Belgium	Caucasian, Asian	Hospital outpatient service	36 ± 9	33 ± 10	109/55	69/48	36/7	4/0
Wojitani, 2012 [15]	Brazil	White/nonwhite	Hospital outpatient service	32 (18–50)	30 (19–49)	100/100	58/84	30/15	12/1

Studies describing association between MBL codon 54 gene polymorphism and VVC									
Liu, 2006 [17]	China	Chinese	Hospital outpatient service	33 ± 8	31 ± 5	51/54^c^	34/43	17/8	0
Giraldo, 2007 [16]	Brazil	White/nonwhite	Hospital outpatient service	31 (18–68)^c^	33 (16–70)	28/66	18/53	10/12	0/1

MBL: mannose-lectin binding; RVVC: recurrent vulvovaginal candidiasis; VVC: vulvovaginal candidiasis.

^a^Expressed as median with range or mean ± standard deviation, as reported in the original paper.

^b^For three of 51 patients, genotypes were not determined because DNA could not be extracted from the respective samples.

^c^Calculated on the overall group of (R)VVC cases, as reported in the original paper.
